# A stakeholder perspective on poverty reduction through the implementation of social marketing in the context of tourism

**DOI:** 10.3389/fpsyg.2023.1304952

**Published:** 2024-01-04

**Authors:** Tuba Türkmendağ, Azize Hassan

**Affiliations:** ^1^Department of Recreation Management, Faculty of Tourism, Atatürk University, Erzurum, Türkiye; ^2^Department of Tourism Management, Faculty of Tourism, Ankara Haci Bayram Veli University, Ankara, Türkiye

**Keywords:** tourism, poverty reduction, social marketing mix, target group, behavior change, exchange theory, phenomenology, grounded theory

## Abstract

This study aims to establish a model that identifies the role of tourism, target group, tourism stakeholders, and the marketing mix factors in the context of tourism marketing based on behavior change and exchange theory, to contribute to poverty reduction through the implementation of social marketing in the tourism context. Phenomenology and grounded theory designs were used in the research. The research findings have revealed that social marketing tools and techniques are effective methods for reducing poverty in the context of tourism and have contributed to a better understanding of the impact of tourism on poverty in terms of roles, barriers, and expectations. It was concluded that for social marketing to be successfully applied in tourism, it needs to be adapted to government policies, institutions, and the private sector. According to the results of the study, government policies should be conducive to promoting behavior change through tourism. In line with the philosophy of social marketing, it is expected that the results will focus on behavior change in the target group in the context of tourism, creating shared values for society, and developing roles for the benefit of individuals and society.

## 1 Introduction

Tourism is the world’s third largest industry with the power to create jobs, stimulate entrepreneurship, develop local economies, and reduce trade deficits, although its negative impacts are still debated from a theoretical and practical perspective. As such, tourism plays an important role in the prosperity of many destinations (United Nations World Tourism Organization, 2020). In the context of this role, tourism is a powerful tool to provide local people with business knowledge and skills (Hadi et al., 2013), to ensure socio-economic development and reduce poverty, especially in developing countries, due to its job and income potential (United Nations World Tourism Organization, 2004b; Scheyvens and Momsen, 2008; Muganda et al., 2010; Yang, 2015).

Despite the positive contributions of tourism to the economies of developing countries, there is a debate about whether tourism will reduce poverty and research that presents two opposing views, both positive and negative. Tourism is thought to perpetuate unequal relations and dependency and lead to inequitable socio-economic development (Scheyvens and Momsen, 2008). Similarly, Ashley et al. (2001) also mention the instability of tourism. On the other hand, they argue that there are two important reasons to focus on tourism in poverty reduction. These are (i) that some characteristics of tourism may be particularly valuable for poverty reduction, and (ii) that discussions and related activities to make tourism more sustainable/responsible will contribute more to poverty reduction if they focus more explicitly on poverty.

Its negative effects felt on a global scale reinforce the idea that poverty is an important phenomenon that needs to be overcome. In addition to the solution proposals determined by academic research on poverty, international organizations set goals by creating action plans at the global level to ensure world welfare. In this context, almost all of the eight goals of the United Nations Millennium Development Goals (MDGs; United Nations, 2015) and 17 global Sustainable Development Goals (SDGs; United Nations, 2019) aim to overcome the direct or indirect effects of poverty. The first sustainable development goal is “No Poverty” and the second is “Zero Hunger.” The first of the eight Millennium Development Goals (MDGs) is “Eradicate Extreme Poverty and Hunger” (United Nations, 2015). The focus is on education (MDG 2, SDG 4), health (MDGs 4, 5, 6/SDGs 3, 6), food scarcity (MDG 1), and environmental sustainability (MDG 7/SDGs 7, 12, 13, 14, 15), develop a global partnership for development (MDG 8, SDG 17), eliminating inadequacies, obstacles and adversities related to economic, cultural, and social justice (SDGs 8, 10, 16), and eliminating gender inequality (MDG 3/SDG 5) overlap with the goal of reducing poverty in all its dimensions.

While recognizing that tourism has great potential to accelerate progress on the SDGs and is relevant to all of the sustainable development goals, the challenges and threats arising from tourism activities are highlighted. SDG 12 “Responsible Consumption and Production,” SDG 13 “Climate Action,” SDG 1 “No poverty,” SDG 4 “Quality Education,” and SDG 8 “Decent Work and Economic Growth” are among the development goals to which the private tourism sector can contribute most through its activities. The sector also has an impact on efforts to achieve other goals indirectly related to poverty, such as advancing gender equality and protecting the environment. However, challenges such as unsustainable consumption and production, as well as mismanagement of natural resources and waste, affect sustainable development goals. Furthermore, tourism’s potential to advance sustainable development is undermined by external threats such as global economic instability, natural disasters, climate change, biodiversity loss, and regional and international security (United Nations World Tourism Organization, 2018).

The economic, social, and environmental issues that tourism focuses on through global development goals have made it inevitable that tourism has a close relationship with poverty alleviation. Moreover, according to Lazer (1969), developments in social issues such as poverty have encouraged managers and academics to become increasingly interested in marketing’s relationship with society. It has been questioned whether marketing principles and practices can be applied in non-commercial areas (Kotler and Zaltman, 1971; Ling et al., 1992). In this context, it has been suggested that the concept of social marketing can play a useful role in the design and delivery of services to enable the poor to use public services more effectively, efficiently and fairly, and to make them sufficiently sensitive to the social, cultural, and psychological characteristics of the poor (Hameed, 2014; Karnani, 2017).

The gain that social marketers hope to achieve from marketing activities is a behavior or situation that is generally considered beneficial and results in a positive outcome (Birkinshaw, 1989; Perese et al., 2005; Donovan and Henley, 2010). In studies that demonstrate the importance of social marketing in reducing poverty in the context of behavior change, the elimination of various addictions, and the harms arising from them (Kikumbih et al., 2005; Messerlian and Derevensky, 2007; Evans and McCormack, 2008; Wymer, 2015), preventing child labor (Edmonds and Schady, 2012), better agricultural practices (Peterson, 2015), improving the quality of health services (Pettigrew, 2016), and the relationship between poverty and education (Tarabini and Jacovkis, 2012). Therefore, the impact of social marketing in reducing poverty in the context of the issues discussed in the literature and its applicability overlaps with sustainable development goals.

The common goals of social marketing and tourism are environmental protection and sustainability (Eagle et al., 2016; Borden et al., 2017; Tkaczynski et al., 2020), sustainable consumption (Hall, 2014), and behavior change (Truong and Hall, 2015b; Borden et al., 2017). However, the multifaceted role of the human factor, which is the determining factor of tourism, in the contribution of social marketing to poverty alleviation has been overlooked. Therefore, limited studies (Truong and Hall, 2013, 2015a) have been found in the literature on this subject. Truong and Hall (2013) analyzed tourism-related projects funded by NGOs in Vietnam according to social marketing benchmarks and found that they can be effective in promoting behavior change for sustainable tourism development and poverty reduction. Truong and Hall (2015a) focused on local people’s perception of poverty, poverty reduction through tourism development, and behavioral change in reducing poverty through social marketing, with a focus on economic wealth creation.

The current study examines the impact of tourism on poverty reduction from a social marketing perspective. In the relevant literature, it is noteworthy that there is a lack of studies with a holistic perspective on whether social marketing in the context of tourism can contribute to poverty reduction and how this contribution can be achieved. Considering the studies on the research area, it has been determined that there are deficiencies in the studies that do not cover all tourism stakeholders and that there are deficiencies in determining the target group and target group-specific social marketing mix. In addition, it is noteworthy that there is a lack of studies that address poverty, social marketing, and tourism in all economic and non-economic aspects and examine the relationship between the concepts. On the other hand, the current study will contribute to the literature in terms of discussing the issue in the context of the gains that the target group will achieve within the scope of the exchange theory and the behavior change expected to be developed. The presence of both positive and negative views on poverty reduction through tourism in the literature is an important reason for conducting the current study. Based on the cautious perspective in the literature, the current study addresses the issue from a perspective that does not accept that tourism will definitely reduce poverty. In this context, the participants in the study evaluated the tourism phenomenon together with its negative aspects and identified the barriers in the context of tourism and the barriers to poverty reduction through tourism from a stakeholder perspective. Thus, a holistic perspective was tried to be reflected in the study by addressing the negative aspects and criticisms in the context of barriers to poverty reduction through social marketing in the context of tourism and positive aspects in the context of roles.

In this study, the role of tourism in reducing poverty is examined within the scope of behavior change and exchange theory used by social marketing and recommendations are presented for practitioners and academics through the model developed. Kotler and Lee (2009) emphasize that exchange theory is one of the effective elements in demonstrating the power of social marketing in reducing poverty. Behavior change, which is defined as stopping, continuing, or encouraging a certain activity performed by the target audience (Dann, 2010), can be an effective method to eliminate poverty in societies (Kotler and Lee, 2009; Lee and Kotler, 2009). Accordingly, social marketing aims to create social impact through behavior change and the application of marketing concepts and techniques to social issues (such as poverty alleviation; Lefebvre, 2013).

The primary objective of the current study is to determine whether the application of social marketing in the context of tourism can contribute to poverty reduction. In this context, in order to contribute to the literature, policy makers, private and public sector practitioners, the study was carried out in line with the following objectives. In addition, the research objectives and the pattern of the study support each other.

- The current study examines the contribution of tourism to poverty reduction and the roles and barriers in this regard from a socio-cultural perspective that encompasses community welfare and development as well as an economic focus. In this context, the study, which focuses on the social marketing perspective, is carried out in a way to cover the views of the actors who play a key role in the tourism sector, which gives the study a unique characteristic.
- By expanding the scope of the subject with the different approaches of all stakeholders, it includes common and contrasting views with a holistic perspective. In this context, determining the views of tourism stakeholders representing a significant portion of local governments, non-governmental organizations, development agencies, tourism enterprises, and local people is the basis of this study.
- The research also benefited from the exchange theory and the behavior change approach, which is the basis of social marketing philosophy. It is possible to explain the manner in which the exchange theory and behavior change are handled in the research as the gains that the target group obtains/will obtain in return for incurring costs/sacrifices in the form of changing their lifestyle, perspective, behavior and activities in social, cultural, and economic terms through the implementation of social marketing in the context of tourism. In this relationship, behavior change itself also plays a role as a gain. The study also tried to identify the target group and responsible stakeholders as the parties of the change that will take place through the implementation of social marketing (change offerer/offerer and change beneficiary/affected) and the marketing mix elements to be used in communicating the change offer.

The aims of the study and will be sought to answer the following Research Questions (RQ) in line with the determined objectives:

RQ1: What is the role of tourism in reducing poverty?
RQ2: What are the barriers to poverty reduction through tourism?
RQ3: How can the implementation of social marketing in the context of tourism contribute to poverty reduction?
RQ4: Who are the stakeholders and target groups in the implementation of social marketing in the context of tourism?
RQ5: What are the elements of the marketing mix in the implementation of social marketing in the context of tourism?

The data obtained in the study were analyzed with NVivo 12. Descriptive analysis and content analysis methods were used together. Based on the research findings, a model proposal was developed and recommendations were presented for both practitioners and academics.

## 2 Conceptual framework

### 2.1 The relationship between poverty reduction and tourism

The definition of poverty has evolved over time from an economic approach to a holistic assessment. In this context, Alagh (1992) defined poverty economically as “being below the absolute minimum welfare level and the inability to satisfy the need for goods and services necessary to sustain life.” The human rights approach, on the other hand, emphasized the multifaceted nature of poverty and drew attention to its socio-cultural dimension (stigmatization, discrimination, insecurity, and social exclusion etc.; Office of the United Nations High Comissioner for Human Rights, 2007). According to Buffoni (2015), poverty should be examined in terms of its macro and micro dimensions as well as its social, cultural, individual, and societal aspects. The psychological context is also an important component of poverty, which includes individuals’ perceptions and emotions in relation to the resources needed for inclusion, full participation in society, and the environmental aspects of their lives (Guagnano and Santarelli, 2016).

It is emphasized that poverty is a problem that not only underdeveloped or developing societies but also developed societies are trying to overcome. According to the Food and Agriculture Organization of the United Nations (2015), 11 million people in developed countries live under the poverty line and face malnutrition. While poverty rates are below 5% in developed countries, it is 12.9% in developing regions. In less developed countries, including Turkey, approximately 512 million people suffered from malnutrition in 2014–2016 (United Nations, 2014). Global Multidimensional Poverty Index evaluates poverty including health, education, and living standards in three dimensions. Accordingly, 1.3 billion people (22%) in 107 developing countries live in multidimensional poverty and half of the multidimensionally poor (644 million) are children under the age of 18 (United Nations Development Programme, 2020). In Turkey, while the share of the poorest group in total income was 6.2% and the share of the highest income group in total income was 47.2% in 2016 (Turkish Statistical Institute, 2016), the share of the poorest group in total income was 6.2% and the share of the highest income group in total income was 46.3% in 2018 (Turkish Statistical Institute, 2019).

Tourism is a complex and ever-growing phenomenon that has become one of the world’s largest economic activities. Earnings from the tourism sector, which consists of many industries such as tour operators, travel agencies, transportation, accommodation, recreation, entertainment, and food and beverage services (Bazini and Nedelea, 2008), is an important source of income for many destinations around the world. However, despite the contribution of tourism to the economies of developing countries, there is a debate about its contribution to poverty reduction. Some researchers have argued that there is not enough empirical evidence that tourism reduces poverty and that the results may be contradictory (Croes and Vanegas, 2008; Goodwin, 2008; Mitchell and Ashley, 2010; Sharpley and Naidoo, 2010; Croes, 2014; Rakotondramaro and Andriamasy, 2016).

Rakotondramaro and Andriamasy (2016) argue that tourism can increase the number of people living on low incomes as well as economic growth, so tourism development increases economic growth but does not contribute to poverty reduction, Croes (2014) argues that tourism will not be effective in reducing poverty in the long run, and Sharpley and Naidoo (2010) state that there is no clear relationship between pro-poor tourism initiatives and poverty reduction, and that traditional tourism practices may actually be more effective in reducing poverty. While the contribution of tourism to employment, economic growth, entrepreneurship, and cross-sectoral linkages is recognized, it is also noted that it can exacerbate inequalities at the international and local levels (Harrison, 2008). Similarly, Ashley et al. (2001) also highlight the instability of tourism and its vulnerability to national and international events, noting that while tourism offers very short-term survival strategies, it can also lead to exploitative employment (e.g., prostitution), weakening human and social capital, and trapping people in long-term poverty.

In addition to concerns about the effects of tourism on poverty in economic terms, the fact that it ignores disadvantaged groups in social, cultural, and religious terms can bring some negativities. Women lag behind men in tourism employment due to religious norms and strong patriarchal structures (Tucker, 2007), and women are deprived of the opportunities offered by tourism due to cultural gender identity, roles, and relationships (Tucker and Boonabaana, 2012), which can lead to gender inequality. Despite tourism’s potential to generate prosperity, developing countries in particular face problems in overcoming poverty, such as colonization, ethnic diversity, and post-colonial tourism activities dominated by multinational tourism companies, and low levels of education (Scheyvens and Momsen, 2008). According to Zhao and Ritchie (2007), as a region becomes richer, the benefits of economic growth will reach the poor through many channels. However, in practice, much less attention has been paid to the extent to which tourism development contributes to poverty reduction.

Criticisms of poverty reduction through tourism by researchers are that tourism aggravates poverty or at least does not bring long-term benefits. Researches that make positive arguments on the subject attribute the success of tourism in reducing poverty to the condition that the gains obtained from tourism are transmitted directly or indirectly to the poor. This situation reveals that both studies that support the negative view and the positive view approach poverty reduction through tourism from a cautious perspective. United Nations World Tourism Organization (2004a) states that tourism can be used as an important force for poverty reduction and emphasizes the need for stakeholders including development agencies, government, and the private sector to work together. In this context, despite the criticisms of researchers that tourism can reduce poverty, a framework has been created and strategies have been determined by international organizations in order to eliminate the negativities. In the context of these strategies;

- Seven action areas have been determined for a tourism development that enables the poor to benefit from tourism. Accordingly; (i) poor people should be employed in tourism enterprises. (ii) Tourism enterprises should benefit from the goods and services of the poor. (iii) Touristic products should be sold directly by the poor. (iv) Tourism enterprises should be established and managed directly by the poor. (v) Taxation in tourism should be done in a way that benefits the poor. (vi) Voluntary actions should be taken by tourism enterprises and tourists. (vii) The poor should benefit from basic infrastructure investments made to develop tourism (United Nations World Tourism Organization, 2004a; World Tourism Organization, 2005). Also, United Nations World Tourism Organization (2010) suggests three main reasons for the importance of tourism in the fight against poverty. First, tourism plays a fundamental role in the economy in many developing countries. Second, the size of the sector and the sustainability of growth play an important role. Third, the characteristics of tourism as an activity are of interest to low-income countries and their poor communities.
- United Nations Development Programme (2011) identified a total of 13 criteria in two groups for increasing direct benefits to the poor. The first group of criteria addresses the requirements for creating the right environment for tourism development. The second group of criteria includes practices necessary for poverty reduction through tourism.
- World Economic Forum (2017) highlighted the need for greater investment in tourism education, public policy-making, and the inclusion of SMEs in the tourism value chain in order to take full advantage of its job creation capacity. In this context, he emphasized that bringing together the public sector, companies and educational institutions can help tourism achieve its goal of reducing poverty. At the same time, tourism should increase its contribution to a fairer society by supporting policies in areas such as entrepreneurship, gender equality, and youth employment.

There are researches in the literature that poverty can be reduced through tourism. For example, tourism contributes to poverty reduction and employment in poorer parts of the world through the expenditures made by tourists traveling to these regions (Spenceley and Meyer, 2012). In particular, low-income countries reduce extreme poverty rates by improving their tourism performance to achieve ideal living standards, accelerate social capital creation, and strengthen sustainable management of natural resources (Kim et al., 2016). Similarly, Kibicho (2003) discussed tourism in terms of its socio-economic impacts such as employment opportunities, personal income, standard of living, prices of goods and services, attitudes towards work, crime, honesty in any commercial exchange, and quality of life. Thus, tourism creates positive effects such as empowering local people economically and psychologically and creating a desire to participate (Yang et al., 2020). Mahadevan et al. (2021) found that poverty reduction in rural areas can be achieved when tourism development policies are successfully implemented. In the fight against poverty, tourism contributes to areas such as education, health, and environment (Kennedy and Dornan, 2009; Mensah and Amuquandoh, 2010), is an important source of foreign exchange in many underdeveloped and poor countries (Blake et al., 2008), creates job opportunities and income for local people, and reduces dependence on international aid (Tabash, 2017).

Tourism contributes to poverty reduction by improving the quality of institutions. Improvements in the quality of institutions in a country affect long-term growth rates. In this context, tourism has strong positive effects on poverty reduction in a country (Zhao, 2021). The importance of protecting and developing local initiatives and small businesses in poverty alleviation through tourism is mentioned. Moufakkir and Kelly (2010) noted that SMEs can reduce barriers to entry and facilitate participation in poor areas, contributing to development and poverty reduction. In addition, tourism cooperatives that support local initiatives contribute economically and socially to poverty reduction by developing local people’s skills/knowledge, creating an environment of equality, prosperity, sense of ownership, democracy and trust, and increasing tourism awareness and education (Yang and Hung, 2014). For example, government subsidies for homestays, businesses providing vocational skills training to the poor, brokering the sale and purchase of tourist souvenirs made by the poor, and improving infrastructure facilities in poor areas will all contribute to poverty reduction through tourism (Qin et al., 2019). Tourism reduces poverty by providing knowledge and diversifying jobs by utilizing the agricultural production of local people (Khan et al., 2020). It has also been shown that tourism supports women’s productivity and the use of their skills (Türkmendağ, 2020) and plays a role in empowering women and promoting gender equality (Alonso-Almeida, 2012; Aghazamani et al., 2020).

The impacts of tourism in line with poverty reduction targets are categorized under three headings; direct, secondary, and dynamic impacts. Direct impacts of tourism on poverty refer to tourism revenues from tourist expenditures, employment, and GDP. Secondary impacts include indirect impacts generated by purchases to provide inputs to tourism enterprises and to meet the needs of tourists, and induced impacts generated by tourism expenditures and business profits. Dynamic effects include infrastructure investments, human capital formation, and the development of other economic activities (Medina-Muñoz et al., 2016). On the other hand, these effects also lead to some negative consequences. The regulation of the coast for tourism activities may hinder activities such as fishing, which is the livelihood of local people, and there may be a conflict with local people due to negative impacts (Mitchell and Ashley, 2010). Truong (2018) states that street vendors improve the living conditions and education of their families, but the type of goods offered, the demand of tourists, competition in the market, and relationships with security personnel that prevent selling in tourist areas lead to a decrease in their income.

Zhao and Ritchie (2007) argue that since poverty has become one of the greatest enemies of humanity in the 21st century, it is worth the research effort and tourism, as one of the biggest drivers worldwide, should play a more active role in poverty reduction. Scheyvens (2011), in her book “Tourism and Poverty,” argues that while tourism can reduce poverty, it should be approached cautiously. It is interesting to note that there are no successful examples that show that tourism activities are successful in bringing long-term benefits and better livelihoods for participants. Jiang et al. (2011), on the other hand, noted that although there is still insufficient evidence that tourism reduces poverty, researchers have been working to provide empirical evidence in recent years. In this context, it is another noteworthy issue identified in the literature review of the current study that empirical evidence on whether poverty can be reduced through tourism has been sought in studies conducted since 2010.

### 2.2 Social marketing in tourism as a tool for poverty reduction

Although the focus has long been on economics, Kotler and Levy (1969) associate marketing with the promotion of arts, culture, education, and health services and the efficient use of natural resources to serve, satisfy, and enrich people’s lives. Accordingly, Kotler and Zaltman (1971) define social marketing as “the use of marketing principles and methods to promote a social cause, idea or behavior.” Andreasen (1994) stated that the main objective of social marketing is to change the attitudes, beliefs, and behaviors of individuals or organizations for a social good and to create social exchange. The definition of social marketing has been developed as the adaptation of technology to programs designed to influence the voluntary behavior of target group (Andreasen, 1994) and to provide solutions to social problems (Hameed, 2014) and the use of commercial marketing principles and techniques (Weinreich, 2011) to improve personal and social welfare.

The focus of social marketing definitions is on “behavior change.” Behavior change enables social marketers to understand why people behave the way they do and what they can do to change that behavior (Maibach, 2003). Moreover, strategies designed to promote behavior change should include the traditional 4Ps of the social marketing mix (Bright, 2000). Accordingly, the social marketing mix should design “attractive benefit packages (product)” and minimize costs (price), make it convenient and easy to shop wherever possible (place), and deliver powerful messages to target group (promotion; Morris and Clarkson, 2009). In addition to the 4Ps, factors related to the personnel who will manage and execute social marketing activities, the development of interpersonal skills, product knowledge skills and process skills, and understanding the needs of the personnel and the target audience refer to the “people” mix in social marketing (Donovan and Henley, 2010). Supporting individuals and organizations refer to the “partnership” mix (Saini and Mukul, 2012), internal groups involved in program approval or implementation, and external groups including the target audience, secondary audiences, and policy makers refer to the “public.” In social marketing, “policy” refers to political changes to promote behavior change, understanding political drivers and constraints, and developing strategies to inform and persuade political actors who can influence the marketing program (Brown, 2006).

In the social marketing approach to poverty, profit is not a priority or a condition, but a socially desirable outcome (Achrol and Kotler, 2016). In addition, social marketing demonstrates its power in poverty reduction through three marketing-specific methods. These are exchange theory, market segmentation, and competitiveness (Kotler and Lee, 2009). Exchange theory involves the transfer of tangible or intangible elements between two or more social actors in the context of social marketing (Hastings and Saren, 2003). Market segmentation refers to the identification of target group among several market segments that require different interventions in order to use scarce resources more efficiently (Morris and Clarkson, 2009). Competitiveness refers to the fact that social marketing offers, although valuable and difficult, are unattractive to the target group, leading people to choose between good and bad behaviors, and that social marketing offers long-term benefits (Hastings, 2003). These competitive conditions specific to social marketing make it difficult to achieve targeted behavior change in poor target group.

Poverty can feed desperation and even lead to crime, and makes people vulnerable to health problems, exploitation due to the hopelessness felt, and a number of negative consequences of illegal migration (Kotler and Lee, 2009). Social marketing, on the other hand, seeks to positively influence the behavior of a society by explaining how the best can be done with the means available, even in societies living in the worst conditions, based on the fact that the economic and non-economic aspects of poverty cannot be separated from each other. Therefore, the contribution of marketing to issues such as climate change, environmental destruction, natural resource scarcity, rapid population growth, hunger and poverty, and inadequate social services has become a subject of research (Serrat, 2017). In this context, Muuka and Choongo (2009) state that the traditional 4Ps can be applied to social marketing in poverty reduction. Thus, social marketing can generate knowledge for the poor as well as improve their ability to make better consumption decisions and to act individually and collectively (Blocker et al., 2013). In the context of behavior change, social marketing aims to break the chain of poverty, improve the quality of human resources, and change behaviors that do not support the welfare of poor communities (Hiqmah, 2017). In the context of the exchange theory, social marketing tries to ensure that the poor adopt poverty-relief behaviors in exchange for poverty maintenance behaviors. Poverty maintenance behavior has low risk and low return, but poverty reduction behavior provides higher returns despite higher risk (Kotler et al., 2006). As mentioned, this situation makes it difficult to be competitive in the context of social marketing.

In this context, the studies discussed include examining the issue of gender equality with a social marketing approach in tourism advertisements (Chhabra et al., 2011), social marketing implementation in entertainment services (Kaczynski, 2008), the benefits and harms of people’s visiting national parks (Wilkinson, 2003), the development of environmentally friendly tourism activities (Kim et al., 2006), behavior change to ensure sustainability in tourism, social marketing measures, technological innovations, emphasizing the need for comprehensive strategies that integrate product innovation, change in transportation model and renewable energy initiatives can be given as examples (Gössling et al., 2008).

It addresses the reduction of emissions from the vehicles tourists use in their travels through behavior change (Peeters et al., 2009), the use of social marketing tools and techniques for a sustainable approach in tourism (Dinan and Sargeant, 2000), and the implementation of social marketing in tourism to encourage environmental behavior of tourists (Tkaczynski et al., 2020). Bright (2000) argues that the use of recreation and tourism activities within the scope of social marketing to provide multifaceted benefits for the benefit of society will help improve the quality of individual and community life. In addition, for social marketing to be effective in the tourism sector, Wearing et al. (2007) stated that (i) visitors should be segmented, (ii) these segments should be associated with varying degrees of sustainable behavior, (iii) the underlying behavioral motivations of these segments (such as the reasons for choosing to visit a particular region and what expectations they hope to achieve) should be understood, and (iv) variables that may have the most tendency to influence visitor behavior should be easily identifiable. Truong and Hall (2015a) investigated the potential of social marketing in tourism development to reduce poverty. According to this study, the most important poverty reduction strategy of social marketing is the need to understand the target group to ensure success in increasing the likelihood of behavior change through well-formed implementation.

It is observed that there are limited studies on the relationship between tourism and poverty from a social marketing perspective. Therefore, this study focuses on identifying solutions to reduce poverty through social marketing in the context of tourism from the perspective of stakeholders. It also aims to identify the target group, marketing mix and responsible stakeholders in the context of social marketing. In poverty reduction through tourism, it is aimed to determine the gains that the target group will provide through the exchange theory and the obligations and sacrifices that will arise from this and to evaluate them in the context of behavior change. It is tried to determine the gains and behavior changes of the target group. It is aimed to determine the role of tourism in poverty reduction and also the barriers that may arise in achieving this goal in the context of behavior change and exchange theory.

## 3 Methods

### 3.1 Research design

In this study, a qualitative method was applied. Qualitative research approaches the world from the perspective of individuals, situations, events, and the processes that connect them, aiming to provide an explanatory understanding of how certain situations and events affect others (Maxwell, 2013). Therefore, in this study, the qualitative research method was used to gain a detailed insight into a complex subject, to amplify the voices of individuals by sharing their stories, and to understand how participants perceive the phenomenon of poverty and what reasons they attribute to poverty within the context or environment they consider (Creswell, 2013). In this context, the study has examined participants’ perspectives on the phenomenon of poverty and the attributions they make regarding the causes of poverty. The study has also aimed to determine the role of tourism in poverty reduction and to identify potential barriers, as well as to elucidate the components of social marketing (target group, marketing mix, and stakeholders) in the context of tourism that would contribute to poverty alleviation efforts.

The study employed both phenomenology and grounded theory approaches. Phenomenology was used to analyze how individuals perceive phenomena (Sart, 2017) and to determine participants’ thoughts and perceptions regarding poverty reduction through tourism. Grounded theory, on the other hand, is an inductive approach that helps make sense of data and develop a grounded framework (Merriam, 2009). Therefore, within the context of tourism, the role of social marketing in poverty reduction and the barriers, as well as the components of social marketing such as the target group, stakeholders, and marketing mix, were determined using grounded theory.

### 3.2 Sampling and research area

The sample of the research consists of individuals representing various stakeholders in the tourism sector, including residents, public and private sector stakeholders in tourism, and non-governmental organizations (NGOs), selected using the maximum diversity sampling method. This sampling method aims to maximize the diversity of individuals who could be stakeholders in the research problem, thereby preventing a one-sided representation of the issue and ensuring that participants’ expressions regarding the problem’s similarities and differences are equally reflected (Patton, 2002). In phenomenological research, the sample should consist of individuals with experience related to the phenomenon, while in grounded theory research, it should include individuals who react to or participate in actions related to the central phenomenon (Creswell, 2013). Therefore, the research aimed to include the most ideal sample group that would meet these requirements. In this context, public sector participants were represented by the Provincial Directorate of Culture and Tourism, the Northeast Anatolia Development Agency, and the university (The university is represented by Atatürk University in Erzurum). Private sector participants were represented by Group A travel agencies, two restaurants with the Ministry of Culture and Tourism operation certificate, one restaurant with historical and touristic importance, as well as four and five-star accommodation establishments in the Palandöken Ski Center. The NGOs were represented by the Erzurum Chamber of Commerce and Industry, the Erzurum Strategic Entrepreneurs Association, and the City and Culture Research Association (See Table 1). The research area encompasses the city center and Palandöken district of Erzurum province, located in the Eastern Anatolia Region of Turkey. Two main factors influenced the selection of the research area. First, Erzurum is one of Turkey’s most famous tourism centers, with intense winter tourism activities on Palandöken Mountain. Second, in 2017, Erzurum ranked 60th among 81 provinces in Turkey in terms of GDP *per capita* on a provincial basis (The Union of Chambers and Commodity Exchanges of Türkiye, 2019).

**Table 1:** Data collection process and characteristics of the participants.

Participants	Participant code	Sex	Age	Education	Occupation	Interview date
Provincial directorate of culture and tourism	PDCT1	M	62	Bachelor’s Degree	Deputy Provincial Director (responsible for tourism)	20.2.2019
Development agency	DA1	F	34	Bachelor’s Degree	Head of Promotion & Cooperation Unit	26.3.2019
University	UN1	M	34	Master Degree	Academician	26.3.2019
	UN2	M	30	Ph.D.	Academician	26.3.2019
	UN3	M	42	Ph.D.	Academician	01.4.2019
	UN4	F	31	Ph.D.	Academician	03.4.2019
Hotel management	HM1	F	30	Bachelor’s Degree	Director of Human Resources	23.1.2019
	HM2	F	27	Two-year Degree	Food Technician	23.1.2019
	HM3	M	46	High school	Chief Accountant	23.1.2019
	HM4	M	40	Bachelor’s Degree	Front Office Manager	06.2.2019
	HM5	M	33	High school	Bars Chief	06.2.2019
	HM6	M	34	Bachelor’s Degree	Director of Human Resources	21.2.2019
	HM7	M	52	Bachelor’s Degree	Food and Beverage Manager	21.02.2019
	HM8	M	34	High school	Housekeeping Chief	23.02.2019
	HM9	M	41	Middle School	Restaurant Chef	23.02.2019
Travel agencies	TA1	F	37	Bachelor’s Degree	Travel Agency Operator	18.02.2019
	TA2	M	37	High school	Travel Agency Operator	18.02.2019
	TA3	F	40	Bachelor’s Degree	Travel Agency Operator	19.02.2019
	TA4	M	38	Bachelor’s Degree	Travel Agency Operator	25.02.2019
	TA5	F	28	Bachelor’s Degree	Travel Agency Manager	25.02.2019
	TA6	M	40	High school	Travel Agency Operator	27.02.2019
	TA7	F	23	Bachelor’s Degree	Sales Representative	01.03.2019
	TA8	M	38	High school	Travel Agency Operator	07.03.2019
Restaurants	RES1	M	52	High school	Restaurant Manager	07.02.2019
	RES2	M	45	High school	Headwaiter	07.02.2019
	RES3	M	31	Bachelor’s Degree	Restaurant Manager	25.02.2019
	RES4	M	55	High school	Restaurant Manager	25.03.2019
NGOs	NGO1	F	36	Bachelor’s Degree	Business and Industrial Relations Specialist	19.02.2019
	NGO2	M	46	Master Degree	Teacher	27.02.2019
	NGO3	M	49	High school	Business Person	27.03.2019
Local people	LP1	M	48	Bachelor’s Degree	Computer Operator	08.02.2019
	LP2	M	20	High school	University Student	14.02.2019
	LP3	M	69	High school	Retired	20.02.2019
	LP4	F	27	High school	Hairdresser	01.03.2019
	LP5	M	48	High school	Self-employment	01.03.2019
	LP6	M	61	High school	Retired	06.03.2019

The ski season in the destination starts in mid-November and lasts until April. The quality of powder snow and the high number of sunny days in the season are important advantages in terms of winter tourism. There are Palandöken Ski Center, Konaklı Ski Center, and Kandilli Ski Center in Erzurum, where winter tourism can be carried out. For winter sports, there are two jumping towers, two jumping ramps and three training ramps, as well as ice sports facilities including ice skating, ice hockey, and curling arenas (Ministry of Culture and Tourism, 2017). With all these opportunities, Erzurum can host national and international sports organizations. Palandöken hosts International Ski Federation (FIS) competitions. Winter Universiade, held in February 2011, were the driving force in the development of the destination. Approximately 100 million dollars have been invested in preparation for the event. With 212 artificial snowmakers, it guarantees snow coverage of 75% of the skiable area at an altitude of 2,200–3,180 m above sea level (Vanat, 2022).

Palandöken Ski Center has nine mechanical facilities, including five chair lifts, one tele ski, two baby lifts, and one gondola lift, with a total carrying capacity of 8,100 people/h. There are 27 ski-runs in Palandöken Ski Center, the total ski-run length is 28 km and the longest ski-run is 12 km. Night skiing is also possible with four illuminated ski-runs. There are areas for activities, daily facilities, restaurants and cafes, and car parking areas (Erzurum Governship, 2023). There are a total of seven accommodation facilities in Palandöken ski resort, including two 5-star, three 4-star, and two with municipality operating certificates. These facilities constitute 35% of the total bed capacity in Erzurum. The destination hosted 138,092 local and 20,065 foreign tourists in the 2017–2018 ski season (December–March). The total number of overnight stays is 241.374 for domestic tourists and 277.112 for foreign tourists, and the average length of stay is 1.8 days for domestic tourists and 2.3 days for foreign tourists. 26% of the total tourists who came to Erzurum in the 2017–2018 ski season preferred ski hotels (Palandöken) for accommodation. The rate of foreign tourists staying in hotels in the ski resort is around 20%. Among the countries from which foreign tourists visit Erzurum during the ski season, Iran comes first (1.5 days), Azerbaijan comes second (1.8 days), Russia comes third (5.5 days), Georgia comes fourth (5.5 days) is located. In addition, tourists from countries such as China (2 days), Bulgaria (1.6 days), Israel (2.7 days), Syria (1.3 days), United States (2.1 days), and Germany (3.1 days) is hosted. Although the majority of foreign tourists visiting the destination during the ski season are tourists from Iran and Azerbaijan, it is noteworthy that their average stay is low. This is due to the fact that tourists from these two countries use Erzurum as a transit route to cities such as Antalya and Istanbul for shopping and sightseeing. In addition, tourists whose primary motivation is not skiing can choose to stay in hotels located in the city center. For the same reason, it is seen that the number of foreign tourists hosted outside the ski season (22,075 of 35,751 foreign tourists in 2017 were hosted outside the ski season) is higher than the number of foreign tourists hosted during the ski season (Northeast Anatolia Development Agency, 2018).

### 3.3 Data collection and participants’ characteristics

In the research, the data collection technique of interviews was employed, and a semi-structured interview guide served as the data collection tool due to the inability to directly observe participants’ emotions, thoughts, ideas, intentions, as well as past experiences, and the absence of a situation that would prevent the presence of an observer (Patton, 2002; Creswell, 2013). When formulating the interview questions for the research, a literature review served as the basis, and insights from a study conducted by Truong and Hall (2015a) were utilized. To conduct interviews with participants, a detailed list of potential participants was first compiled. Each participant was then contacted by phone to schedule an appropriate date and time for the interview. Interviews were conducted in person at the location where the participant was present, with a focus on ensuring a quiet environment and preventing interruptions by third parties. To avoid missing details during the interviews and expedite the process, audio recordings were made. Before commencing the interviews, each participant was informed about the ethical principles (Baş and Akturan, 2017). This included providing participants with information about the research topic, purpose, the use of audio recordings during interviews, and assuring them that no personal or organizational information beyond their views and thoughts on the topic would be requested. Additionally, it was communicated that audio recordings and the provided information would not be used, shared, or disclosed publicly and that participation in the interview was voluntary.

The data for the research were collected between January 23 and April 3, 2019. A total of four four- and five-star hotels located in the Erzurum Palandöken Ski Center were listed, and following the interviews, participants from four of these hotels agreed to participate in the interviews. However, participants from one hotel establishment could not be included in the research due to work commitments. A total of 34 Group A travel agencies were listed, and 11 of them could not be included in the research due to work commitments. Communication could not be established with five of them due to phone and/or address changes, while 10 agencies directly refused to participate, and eight travel agency participants were included in the research. A total of four participants from three restaurants, including two tourism-licensed restaurants operating in Erzurum and one restaurant with historical and touristic significance, were included in the research. The selection of participants in line with the research’s purpose was also considered during this process, following the requirements of the phenomenological and grounded theory approaches. Phenomenological research can include a sample size ranging from 1 to 325 individuals, but it is recommended to work with 5–25 individuals. Grounded theory research typically involves working with 20–30 individuals, but this number can be higher (Creswell, 2013). Other approaches used to determine sample size include data saturation, which refers to the point where new data contribute little to existing knowledge, and data adequacy, which means that the obtained data are sufficient for making comparisons, interpretations, and explanations (Baş and Akturan, 2017).

In this context, a total of 39 participants were involved in the research, and analysis was conducted using the interview data from 36 participants. The reason for excluding three interviews from the analysis is as follows: technical issues with the audio recording in one interview, unproductive responses from the participant in one interview, and constant interruptions during the interview due to the participant’s work environment in another interview. In addition to audio recordings, observation and research notes were taken after each interview, recording details of the process. These notes were used during the analysis. The average duration of the interviews was 54 min and 50 s. Detailed information about the data collection process and participant characteristics is provided in Table 1.

### 3.4 Data analysis

In the research, the data preparation and analysis involved the transcription of interview recordings, examination of observation/research notes, and the use of the NVivo 12 computer-assisted qualitative data analysis program. A combination of descriptive analysis and content analysis methods was used in the research. The research data, consisting of a total of 420 pages (155,098 words), were reviewed seven times. Within the scope of descriptive analysis, “themes,” and “categories and codes related to the themes” were determined based on the literature and interview questions. Additionally, themes, categories, and codes derived from participants’ responses were also included in the research. Content analysis was employed to identify elements related to the role of social marketing in reducing poverty through tourism. Coding was performed to categorize and theme the data. During this process, data were continuously compared to identify similarities and differences, leading to categorization based on these findings (Merriam, 2009). Non-applicable data were removed following the framework created at this stage (Creswell, 2013). Subsequently, the findings were identified, supported with quotations, and the process involved interpreting, analyzing, and relating the findings.

The coding process was carried out by the first author of the study using thematic coding and axial coding. Thematic coding involves coding the data according to predefined categories and themes. In this context, the views of a diverse group of participants on specific phenomena, objects, actions, events, or ideas were examined. Different codes, categories, and themes that emerged during the coding process were also included in the analysis. Thus, the focus was not only on counting specific expressions in the dataset but also on identifying implicit ideas (Jnanathapaswi, 2021). With axial coding, codes were determined to create a list of codes, leading to the identification of categories and themes. This process was conducted dynamically through continuous comparisons of data as each interview transcript was analyzed (Corbin and Strauss, 2014). The codes obtained were reviewed by the second author of the study, evaluating them for similarities, differences, and alignment with categories/themes. For codes where a consensus could not be reached, input was sought from two academics through peer review, as part of research reliability (Pandey and Patnaik, 2014). Additionally, the coding process was completed by using the var*iation method* (Pandey and Patnaik, 2014) as part of research credibility (internal validity), drawing from observation and research notes taken during data collection.

### 3.5 The validity, reliability, and ethical principles of the research

In ensuring the validity and reliability of the research, four criteria were employed (Pandey and Patnaik, 2014); *credibility (internal validity)*, *transferability (external validity)*, *dependability (reliability)*, and *conformability*. Accordingly, the credibility of the research was established as follows: *(i) Long-term interaction* (considerable time was spent in the field before, during, and after the data collection process, allowing for the observation of participants and the environment). *(ii) Continuous observation* (participants were observed continuously to gather in-depth data and establish effective communication for understanding their perspectives). *(iii)* Var*iation* (in addition to interviews, data diversification was achieved through observations, interviews, field notes, and their utilization in the analysis phase). *(iv) Participant controls* (after analyzing voice recordings, the majority of participants were interviewed in person to confirm their statements, although confirmation could not be obtained from four participants who were out of the city during the data confirmation period). For the transferability (external validity) of the research, *detailed descriptions* were provided (including comprehensive information about the data collection process, instruments, and the sample). The dependability (reliability) of the research was ensured through *peer review* (two academic colleagues specializing in tourism were involved in reviewing the data collection, analysis, and interpretation processes). The confirmability of the research was established through a *detailed account of the research process* (the methodology section of the study provides a comprehensive description of all phases of the fieldwork and written documents have been archived).

Following the ethical principles of the research (Creswell, 2013), the research design process involved obtaining approval from the Gazi University, Social Sciences Institute Ethics Committee under Protocol Number 77082166-302.08.01 and Research Code No. 2018-325. At the beginning of the research, participants were informed about the research’s purpose and how the data would be used. Written and verbal consent were obtained, a safe environment was created for data collection, and care was taken to ensure that the research process was fluid. Leading questions and the expression of emotions were avoided. Each participant was assigned a code to protect their anonymity. During the data analysis process, every positive, negative, and contradictory result was reported, and honesty was maintained in reporting the findings.

## 4 Findings

In this section, themes, categories, and codes obtained through descriptive and content analysis methods have been presented, along with quotations from the participants’ statements.

### 4.1 Dimensions of poverty, the role of tourism in poverty reduction, and perceived barriers

In the context of participants’ assessments of the poverty phenomenon and its causes, the role of tourism in poverty reduction and encountered barriers were examined. In this context, the dimensions of poverty, the role of tourism in poverty alleviation, and encountered barriers were elucidated through the descriptive analysis method (Table 2).

**Table 2:** Dimensions of poverty, the role of tourism in poverty reduction, and perceived barriers.

Themes	Categories
Dimensions of poverty	Economic
	Socio-cultural
	Psychological
	Individual
	Moral
The role of tourism in poverty reduction	Direct effects
	Secondary effects
	Dynamic effects
Perceived barriers to poverty reduction through tourism	Economic
	Political and legal
	Socio-cultural
	Other

#### 4.1.1 Dimensions of poverty

Approaching poverty solely from an economic perspective will be limited in generating solutions to the globally significant issue of poverty. Therefore, the research has focused on presenting not only the economic dimension of poverty but also its other dimensions. The majority of participants primarily evaluated poverty from an economic standpoint. However, they associated the concept of poverty, which they critically examined, with various concepts such as education, culture, values, resilience, work, outlook on life, and expectations. In this context, based on the relevant literature and study findings, the dimensions of poverty were categorized as *economic*, *socio-cultural*, *psychological*, *individual*, and *moral* through the codes obtained.

#### 4.1.2 Perceptions regarding the role of tourism in poverty reduction

The categories obtained through codes regarding the role of tourism in poverty reduction are discussed in the literature as *direct effects*, *secondary effects*, and *dynamic effects*. Regarding the *direct effects* of tourism, it is emphasized that tourism generates *employment* and provides *income to various segments of the population through the expenditures made by tourists in the destination*. It is noted that there will be gains such as *cultural interaction and development* through communication with tourists and *a positive change in the perspective of the local population*. Concerning *secondary effects*, *the economic relationships of tourism with different industries* were evaluated. Within the scope of *dynamic effects*, participants emphasized that *investments in tourism would contribute to meeting the needs of the local population* and that *income generated from tourism could be transferred to the poor*, thus reducing poverty.

If we can guide tourists correctly, create a welcoming environment for people to come comfortably, and direct tourists outside as well, then tourism can thrive. It contributes to the economy, and it benefits both the well-being of individuals and the region as a whole… We need to bring tourists outside, and we need to open up opportunities for production… (TA2).

When I think of tourism, I think of culture. As culture increases, one’s perspective on the world changes. It leads to self-improvement and productivity. When you convey this to people, it becomes a form of tourism diplomacy. It also prevents poverty, all forms of poverty… (TA1).

#### 4.1.3 Perceived barriers to poverty reduction through tourism

In addition to positive perspectives on the contribution of tourism to poverty reduction, some views highlight its negative aspects, deficiencies, and barriers. Based on participants’ thoughts and experiences, barriers encountered in poverty reduction through tourism were categorized as *economic barriers*, *political and legal barriers*, *socio-cultural barriers*, and *other barriers*. Participants predominantly emphasized *political and legal barriers*. It is believed that the majority of participants working in the tourism sector may have influenced the emergence of such a finding.

##### 4.1.3.1 Economic barriers

It has been mentioned that intense tourism activities in the region may *lead to an increase in the prices of goods* and *negatively impact the purchasing power of the local population*. Perceptions of barriers to poverty reduction are primarily focused on the *inability of the local population to integrate into tourism activities* and *the dominance of large businesses in profiting from tourist income-generating activities*. Similarly, it has been expressed that *due to cheap hotels and all-inclusive systems, the local population cannot benefit sufficiently from tourist expenditures*.

Today, when we sell a hotel, the commission we receive is 3%… When we look at the system here, to be honest, the biggest share is taken by the big companies. As intermediaries increase, as brokers increase, the income obtained decreases a bit… (TA6).

##### 4.1.3.2 Political and legal barriers

*Lack of vision, inadequate planning, inability to act together*, and *a focus on short-term plans* are seen as problems stemming from management. *Terrorism* and *the inability to invest in insecure areas* are significant barriers as well. Due to Turkey’s geographical location, it is close to geographies where hot international conflicts occur and the research area is located in the region where terrorist or provocative activities are relatively intense, terrorism is perceived by the participants as a barrier to tourism reducing poverty. It is seen as an element that creates concern among tourists about the safety and security of the destination and negatively affects its image.

…So, it means we have issues with our awareness, we lack a tourism vision, and we have a knowledge gap. After rehabilitating these areas, there is no reason not to generate income, or expectations can also be met if they are not too high. There is no reason for the local population not to participate in a more equipped manner with more rational expectations and gain in the medium and long term… (DA1).

##### 4.1.3.3 Socio-cultural barriers

*The lack of a productive mindset, cultural differences, concerns that tourists will negatively impact the local culture, and the lack of belief and awareness in the benefits of tourism activities* are some of the reasons why *the local population may not readily accept tourists*. Consequently, *the full potential of tourism may not be realized*, which can be seen as a significant barrier to reducing poverty through tourism.

To reduce poverty, one must be productive. To be productive, one must be able to look at things from multiple perspectives and have a multifaceted view of the world. For example, many people in Erzurum have never been skiing. They see the snow not as a source of joy but as a hardship. So, the productivity of turning it into entertainment cannot be imagined… (RES1).

##### 4.1.3.4 Other barriers

Failure of *businesses to meet the workforce needs of the local population, issues related to education*, and *inadequate promotion* are significant barriers to poverty reduction through tourism.

We need to guide tourists. If we can satisfy a guest, they will also guide their surroundings very well. We just need to do more than our best, do good promotion, and advertise well… (HM8).

### 4.2 The role of social marketing in poverty reduction in the context of tourism

The literature has shown that adapting the traditional marketing mix to social marketing, as well as the methods, stakeholders, and target groups of social marketing, can vary depending on the application area. However, there is a lack of a comprehensive model that demonstrates the contribution of social marketing in reducing poverty through tourism. In this context, the study identified the role of social marketing in the context of tourism, target groups, stakeholders, and marketing mix with the help of grounded theory. Through the analysis of the data, categories were reached, and these categories were combined to determine the theme (Table 3).

**Table 3:** Results of content analysis.

Theme	Categories	Codes	*N* ^*^
Implementation of social marketing in the context of tourism	(1) Implementing social marketing in the context of tourism	Awareness raising	14
		Increasing empathy-communication ability	11
		Getting to know different worlds, opening your horizons	10
		Avoiding bad habits	10
		Building intercultural tolerance	9
		Developing the local population’s skills	8
		Increasing production efficiency (local population)	8
		To ensure the social development of the local population	8
		Socialization	8
		People respect each other	6
		Teaching/Learning	6
		Waste management, recycling	6
		Protection of the natural environment-sensitivity to the environment	6
		Creating national consciousness	4
		Creating a culture of sharing	3
		Understanding the value of life	3
		Rehabilitation	3
		Increasing motivation	3
		Moving away from terrorism and similar activities	3
		Efficient use of resources	3
		To ensure the development of tourism employees	2
		Gaining self-confidence	1
	(2) The role of social marketing in reducing poverty in the context of tourism	Encouraging the local population to use their energy wisely/directing them towards productive activities.	17
		Developing a positive outlook in the local population	10
		Ensuring that the local population stays away from bad habits	9
		Increasing personal development	8
		Interaction between cultures	7
		Changing the lifestyle	7
		Conscious use of resources (prevention of waste)	5
		Revitalizing-preserving local values	5
		Obtaining a profession	4
		Positive reflections on the tourist profile	3
		Increasing respect	3
		Economic empowerment	3
		Increasing sharing	3
		Mental relaxation/occupying the mind	2
		Increasing the hope and problem-solving skills of the local population	2
		Reducing gender inequality	1
	(3) Stakeholders	Local administration	20
		Local population	18
		Tourism businesses	16
		NGOs	13
		Government/relevant ministries	12
		Governorship	11
		Universities	11
		Provincial tourism directorates	7
		Ministry of National Education/educational institutions	6
		Culture and Tourism Ministry	5
		Local businesses	3
		Various (related) professional groups (sociologist-psychologist)	3
		Professional associations related to tourism	3
		Tourists visiting the destination	1
		Cooperatives	1
		Media-Press (promotional activities-sponsorship)	1
	(4) Target group	Local population	28
		Children	9
		Youths	9
		Convicts	2
		Disabled people	2
	(5) Social marketing mix	People	32
		Product	32
		Place	24
		Partnership	24
		Policy	12
		Price	11
		Promotion	11

^*^The number of codes repeated by the participants.

In the implementation of social marketing in the context of tourism, the first and second categories, along with the codes grouped under them, have been examined in the context of the exchange and behavioral (behavior change) theories that form the basis of the research. These two categories explain the gains that the target group will achieve through behavior change in the context of tourism through the change theory. In this context, the target group for whom behavior change will be created, the responsible stakeholders for achieving this, and an effective marketing mix have been identified.

#### 4.2.1 The effects of implementing social marketing in the context of tourism

Participants have expressed that implementing social marketing in the tourism context can lead to behavior change within society. In this context, some participants have shared examples based on their own experiences regarding the implementation of social marketing in the sector. Furthermore, it has been emphasized that this effort should be *sustainable, requiring long-term, meticulous work*, and *stability*.

In the end, you win people over, help them abandon bad habits, enable them to express themselves better…, and perhaps enhance their ability for empathy and communication… (RES3).

For example, last year, we conducted a collaborative project with the university’s sociology department. On International Women’s Day, we provided a wonderful day for mothers and children. Thanks to the support of the district family and social policies directorates affiliated with the governorships, transportation was provided… Some families had never visited Erzurum before, and they returned very happy; it was a cultural event for them. This is culture, richness, and tourism. In other words, wherever you guide people, it comes back to you and shapes the child (TA1).

Only a small minority of participants (five participants) expressed that the implementation of social marketing in the context of tourism might not be feasible. Those who held this viewpoint pointed out that *a significant portion of stakeholders in the tourism sector consists of profit-driven businesses*, and *their primary goal is to generate profits*, which they believed contradicts the non-financial benefits characteristic of social marketing. Additionally, some participants expressed concerns that *the government may not prioritize or allocate sufficient time for implementation*, leading to doubts about its feasibility. Consequently, it was noted that implementing social marketing in this context could be challenging.

In this regard, voluntarism is essential. When I think about it, it would burden the government? You would have to employ many people. I don’t think it would be very successful because it doesn’t generate profit. I don’t believe there could be such an example in tourism (LP1).

I don’t think it would happen. The organization’s image would improve and get better, so I think they would consider their benefit when doing this… It’s a beneficial practice for themselves. They may not make money directly from what they do, but they do gain a lot. We should especially emphasize this point in the relationship between social marketing and tourism (UN4).

#### 4.2.2 Reducing poverty through the implementation of social marketing in the context of tourism

Participants who expressed that the implementation of social marketing in the tourism context is possible have stated that it would contribute to reducing poverty in all its dimensions. Thus, it has been noted that this would lead to positive *behavior changes* within the target group.

Indeed, as people’s personal development increases, their lifestyles change, and their perspectives change, most importantly. All of these changes have a positive impact. This positive impact reflects on tourists. Tourist behavior also affects the local population there… (HM6).

One participant who expressed the opinion that social marketing cannot be achieved through tourism has stated that it is not possible to reduce poverty in this way.

So, in my opinion, poverty is not something that can be reduced in this way, except for a few lucky individuals who might succeed. Apart from that, those who are poor tend to remain poor. I don’t think there is a strong correlation between these factors… (LP2).

#### 4.2.3 Stakeholders

Stakeholders are one of the relevant parties alongside the target group in the context of *exchange theory* in this study. The findings of this study indicate that the *exchanges* in the benefits proposed by stakeholders for the target group to create *behavior change* will occur between these two parties. Therefore, stakeholders are a crucial component of the model. Regarding stakeholders, the participants have reached a consensus that the government should take a leading role with all its organs and be able to bring all stakeholders together. It was expressed by the participants that all stakeholders should fulfill their responsibilities, and only through this cooperation, success can be achieved. The role of local governments as implementers of state domestic policies in this regard was particularly emphasized.

We talk about the private sector, but the entity best suited to excel in this is the government. The coordinating entity should be the government, but stakeholders can include the private sector, private foundations, associations, and non-governmental organizations; all of them can be a part of this. However, the government should be the one to outline the main strategy and do the planning… because private enterprises tend to think in an economically focused manner… Our people are very sensitive to such matters, so just as I firmly believe the government should be involved, the public should be involved as well… Universities should also be part of it in the scientific aspect… (UN1).

#### 4.2.4 Target group

Participants expressed their concerns that, due to the impact of poverty in the region, children and young people may be targeted by illegal groups carrying out provocative/terrorist activities and may be more easily persuaded. Participants have expressed that social marketing programs in the context of tourism could be implemented *to engage and rehabilitate young people and children who may have links to terrorism*. They believe that *by ensuring the sustainability of these programs*, negative situations can be eliminated before they occur. Additionally, participants have noted that this approach could *contribute to the formation and enhancement of national feelings and national consciousness*.

*For instance, we talked about terrorism. This is my idea: the municipality and the governorship should, without any discrimination, take children and young people out, show them around, and make them understand that Hakkâri* (A city in the northeast of Turkey) *alone is not their homeland*… *Istanbul is also your homeland… This is something that contributes to the sociology of people (HM3)*.

Some participants, on the other hand, have expressed that social marketing programs could be developed *for convicts and individuals with disabilities*, suggesting that behavior changes can be instilled in them through such programs, ultimately benefiting both society and the individuals themselves. Additionally, within the context of findings related to the target group, social marketing differentiates from social tourism at a certain point. This is because social tourism does not have the primary goal of creating behavior change.

For example, we can direct animation and recreational activities towards disadvantaged groups, autistic children, and children who are marginalized by society… Being with their peers and people in similar situations, and seeing others like themselves, will empower them and create awareness. Furthermore, some habits can be instilled in these children through activities such as games. Games will provide them with self-confidence and instill a sense of “I can do it”… (UN2).

#### 4.2.5 Social marketing mix

Although various approaches have been put forth regarding the social marketing mix, researchers unanimously agree on the four fundamental marketing mix elements: *product, price, place*, and *promotion*. However, the marketing mix to be used in the implementation of social marketing in the context of tourism has not been determined. In this context, the social marketing mix has been determined based on the experiences and opinions of the participants. The results of the research align with the study of Donovan and Henley (2010) which determined the marketing mix. Due to its effectiveness in creating *behavior change* in the target group (Gordon, 2012), the social marketing mix is an important component of the model.

Participants have expressed their thoughts on who should be reached and how in the implementation of social marketing in the tourism context, as well as who should take on roles and responsibilities while referring to the elements of the marketing mix. In this context, the marketing mix elements identified in the study align with the traditional marketing mix: the *product* directed towards the target group, the cost of the product (*price*), the most effective way to reach the target group (*place*), and the efforts (*promotion*). Additionally, the individuals who will design, coordinate, and execute the initiatives, as well as the *people* who will be affected by and benefit from the offered product, collaborative individuals and organizations (*partnerships*), *policies* that will create a strategy to bring all these together are important elements of the marketing mix in the reduction of poverty through the implementation of social marketing in the context of tourism.

…You can do this in terms of advertising with very small budgets… How? For instance, you can make an announcement. Local advertising is very easy… You can use sponsors… You can advertise the event day and time on billboards… (TA3).

## 5 Discussion

The pursuit of equal living conditions and well-being for individuals in society has become a paramount goal for societies. Despite the cautious approaches and even criticisms of some researchers, the number of studies demonstrating that poverty can be reduced through tourism is not insignificant. Indeed, this study has supported the existing research in the literature that suggests tourism can contribute to poverty reduction. In this context, the findings indicate that tourism has a direct, secondary, and dynamic impact on poverty reduction, which is consistent with studies in the literature (Blake et al., 2008; Mitchell and Ashley, 2010; Mitchell, 2012; Spenceley and Meyer, 2012; Winters et al., 2013; Manwa and Manwa, 2014). Additionally, participant opinions have shed light on the role of tourism in poverty reduction and the barriers that may be encountered. The key results regarding the role of tourism in poverty reduction are as follows:

- Tourism particularly increases local employment opportunities for the local population.
- Tourism contributes to individuals’ development by providing them with various skills.
- Through the supply of goods and services, tourism generates economic gains for other sectors.
- Through non-economic gains, tourism contributes to combating poverty in all its dimensions.

The notable research findings on perceived barriers to poverty reduction through tourism are as follows:

- Security issues that may arise at the destination.
- Negative consequences arise from relations and crises between two countries.
- Failure of production, and weak competitiveness in fields such as industry, technology, and science.
- The inability of the local population to benefit from tourism due to the all-inclusive system.
- Emergence of negative attitudes and behaviors in local-visitor interactions due to socio-cultural differences.

In this context, the barriers emphasized the most by the participants fall into the categories of political and legal barriers (such as war, terrorism, chaos, etc.) and socio-cultural barriers (including theft, muggings, begging, harassment, the inability of immigrant/refugee populations in the destination to adapt to the local culture, causing discomfort to tourists, poor living conditions, etc.). Similarly, Uzun (2018) noted that unwanted behaviors can emerge with migration, while Yıldırımalp (2018) mentioned that poor working conditions and the challenges associated with migration can increase the risk of poverty. Participants criticized the all-inclusive system while also highlighting reasons for its imposition, such as the limited number of entertainment venues at the destination, restricted alcohol sales, inconvenient and costly transportation to the city center, and a perception that it is not worth the time. Similarly, Ashley and Roe (2002) pointed out that the tourism industry, often controlled by established businesses, limits entry opportunities for the poor and small (local) businesses. In this context, Bozorgaghideh and Beegam (2014) stated that tourism activities should benefit the poor, tourism destinations should be managed with the aim of reducing poverty, how tourism income is distributed and who benefits from it should be known.

**Figure 1:**
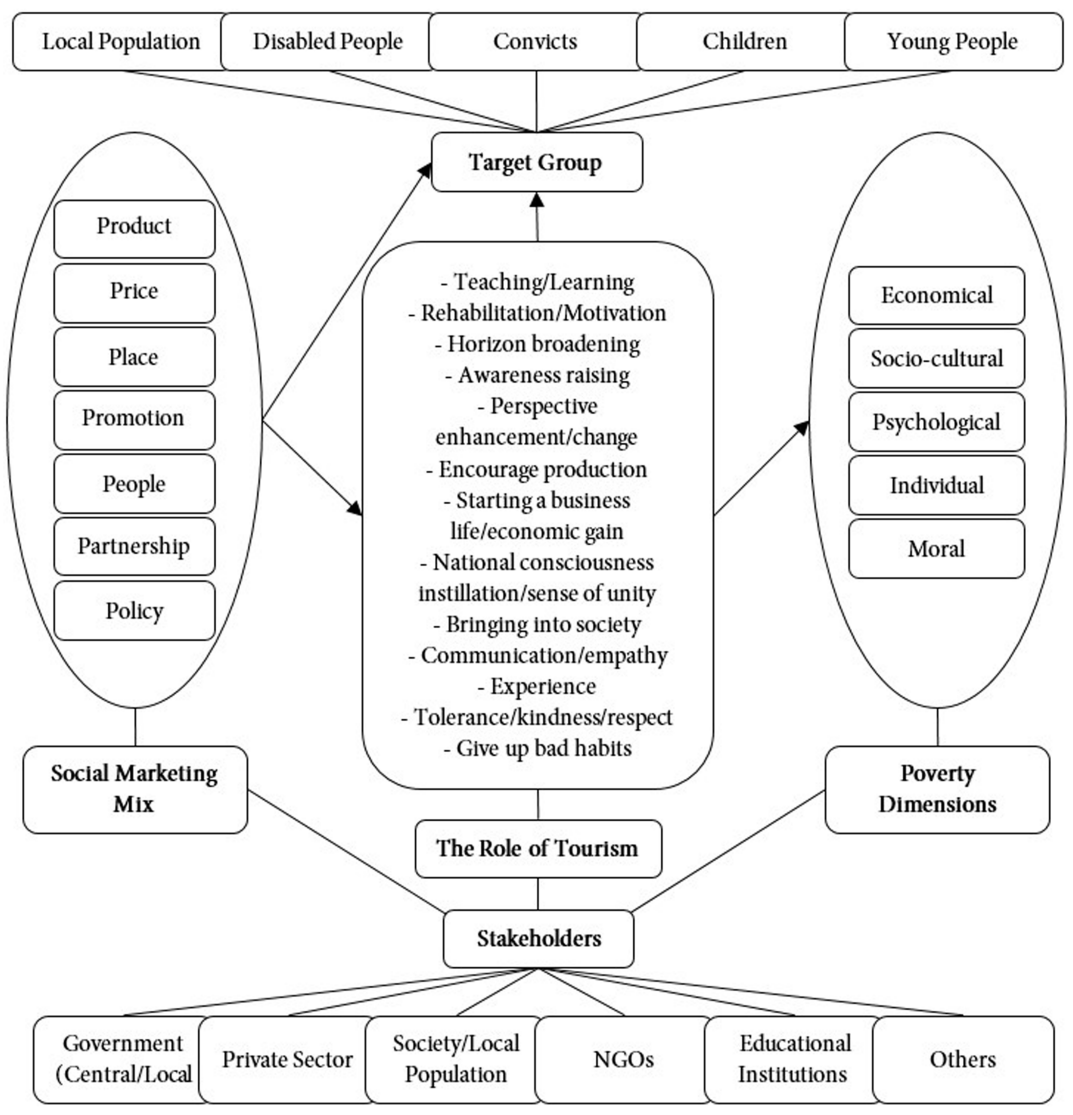
The model of social marketing approach for reducing poverty in the context of tourism.

Gordon et al. (2016) have stated that the idea of using social marketing for social well-being has been discussed for a long time, but in recent years, various discourses, approaches, and perspectives have emerged. In this context, there has been little focus in the literature on implementing the social marketing approach to address poverty reduction in all its dimensions in the context of tourism. However, studies indicating that efforts to reduce poverty through tourism are not solely focused on economic aspects support the contribution of social marketing to poverty reduction solutions in the context of tourism. In this context, the efforts to reduce poverty through tourism have been evaluated with their contributions to areas such as education, health, and the environment (Mensah and Amuquandoh, 2010); the enhancement of local skills, the development of local infrastructure, the involvement of the poor in production, and the preservation and sustainability of natural and cultural resources (Bolwell and Weinz, 2008). The results of the research allow for a focus on the gains and *behavior change* that will arise within the framework of *exchange theory* through the implementation of social marketing in the context of tourism. The categories of *the effects of implementing social marketing in the context of tourism* and *the role of implementing social marketing in the context of tourism in reducing poverty* explain how this behavior change will occur and which gains it will provide. In this research, the identified *target group, stakeholders*, and *marketing mix* play a supportive role in both creating and encouraging behavior change and determining the recipients of the resulting gains. In this regard, the model presented in the study (Figure 1) provides a comprehensive structure in the context of the research purpose. In this context, this research has determined the target group and which behavior change can be developed based on the target group’s needs. The results obtained from the research are as follows.

- *Local population*: Changing the perspective on tourists and tourism, creating awareness, establishing a peaceful and harmonious environment throughout society, promoting active participation in tourism, enhancing individuals’ mental and psychological well-being, fostering communication, preserving values, supporting women entrepreneurship, acquiring new skills, and converting skills into income.
- *Disabled people*: Preventing self-isolation or social exclusion, promoting socialization, realizing that their situation is not unique, empowering, acquiring certain habits through drama, and gaining self-confidence.
- *Children and young people*: Influencing their future decisions, expanding their horizons, rehabilitation, socialization, spending time with peers, becoming part of the community, overcoming addictions, developing nationalistic feelings, and moving away from provocative activities.
- *Convicts*: Becoming a part of the community, utilizing leisure time, clearing one’s mind of negative thoughts, psychological well-being, and turning skills into income.
  Regarding stakeholders, some of the participants have emphasized that institutions and organizations involved in such projects should be voluntary. This finding aligns with the non-profit nature of social marketing implementations. Additionally, participants have highlighted those stakeholders may vary depending on various characteristics of the project, such as its size, purpose, form, and target group. The results regarding primary stakeholders are as follows.
- *Government and relevant ministries*: The government should be at the center and ensure coordination among institutions and individuals. By “government,” what is meant are the relevant ministries, central, and local authorities.
- *Private sector*: Accommodation establishments, travel agencies, food and beverage establishments, recreation and entertainment businesses, and local enterprises have been evaluated within this scope.
- *Educational institutions*: Universities, national education directorates, and students are all stakeholders. Additionally, students can be considered both stakeholders in project implementation and the target group.
- *Non-governmental organizations*: It has been stated that it is both impossible and unnecessary to include all NGOs. However, NGOs that are relevant to the subject and can contribute are seen as stakeholders. Due to their active role in social issues and their more flexible structure compared to other institutions and organizations, they are considered important stakeholders. In the context of a social marketing program within the tourism sector, NGOs can contribute to poverty reduction by assuming roles such as creating awareness, increasing societal participation, supporting efforts for a more equitable society, creating/supporting socio-cultural and economic development projects, encouraging policymakers, building networks, and fostering collaborations. They can also contribute to the social marketing mix by creating or participating in the creation of products.
- *Local population (people-society)*: It has been indicated that regional support can best be provided by the local population, and individuals who are regarded as opinion leaders within the community should be particularly involved in the process. It has been emphasized that projects not embraced by the community are likely to fail, will not receive support, and may even lead to hostility within the community.
- *Other stakeholders*: Volunteer representatives, media, local press, professional associations related to tourism, foundations, non-profit associations, cooperatives, sociologists, psychologists, and healthcare institutions are involved in this context.

The participants who expressed their views on the feasibility of implementing social marketing in the context of tourism also made references to the marketing mix elements that social marketing would use within this framework. In this context, the social marketing mix created is as follows:

- *Product* refers to presentations that will provide benefits to the target group and lead to behavior change.
- *Price* refers to the cost incurred during the design of the product and its delivery to the target group.
- *Place* refers to the methods and practices for delivering the product to the target group or directing the target group to the product (event, organization, project, etc.).
- *Promotion* refers to efforts to inform the target audience, promote the product/event, and enhance its attractiveness utilizing elements from the promotion mix.
- *People* refer to all individuals involved in the entire process from the design of the product to reaching the target group and observing the results, including those responsible for various tasks and the target group itself.
- *Partnership* refers to the stakeholders who will support and collaborate on the project.
- *Policy* involves the development and implementation of strategies that guide all stakeholders and the target group toward the goal through a comprehensive approach.

The elements of the social marketing mix obtained in the research exhibit both similarities and differences compared to the social marketing mix described in the literature. Social marketing largely draws from the elements of the traditional marketing mix. In previous studies, the social marketing mix has been approached primarily based on the components of *product, price, place*, and *promotion* (Kotler and Zaltman, 1971; Maibach, 2003; Wood, 2008; Cheng et al., 2011). In addition to the 4Ps, the unique nature of social marketing has led to the inclusion of different components in the mix. In this context, factors such as *policy* (Brown, 2006; Donovan and Henley, 2010; Luca and Suggs, 2010); *public* (Brown, 2006); *people* (Donovan and Henley, 2010); *partnership* (Donovan and Henley, 2010; Luca and Suggs, 2010; Saini and Mukul, 2012); and *positioning* (Saini and Mukul, 2012) are addressed. Gordon (2012) has outlined the elements of the social marketing mix as conditions, organization and competition, cost, consumer, process, channels, and strategies. In the present research, the proposed social marketing mix is seen as an important guiding and supporting element in the coordination and direction of efforts to reduce poverty through tourism. According to Gordon (2012), the social marketing mix not only provides additional tools for use in behavior change but also incorporates the strategies that are currently in use into the process.

## 6 Conclusion

This research aimed to determine the contribution of implementing social marketing in the context of tourism to reduce poverty. In this context, it focused on examining the perspectives of tourism stakeholders by utilizing their experiences. Conducting the study with a social marketing approach allowed for the incorporation of exchange and behavioral (behavior change) theories that underlie this concept. These theories guided the identification of which components should be included in the model and their contributions. In this regard, previous research has supported the idea that creating behavior change in the target group would be a sustainable way to reduce poverty. Additionally, focusing on benefits and gains as the focal point of the exchange theory will encourage individuals and communities to strive for improvement. Therefore, it is anticipated that sustainable behavior changes will emerge in conjunction with exchange, and this in itself will be a gain.

Only a few participants stated that the primary goal of businesses in the industry is to make a profit and that this is not related to reducing poverty in the context of tourism. On the other hand, it was emphasized that when implementing social marketing in the context of tourism to reduce poverty, it is necessary to focus on long-term goals, as results may not be observed quickly, and there may be difficulties due to the possible existence of conflicting individuals or groups in society. The approaches discussed in the current research will be effective in reducing poverty in all its dimensions, particularly by addressing issues related to the community and the environment in the tourism context. Therefore, it is possible to implement social marketing in the context of tourism as an effective tool to contribute to the establishment and sustainability of social justice and social welfare. Social marketing implementations are an important step in encouraging individuals to develop sensitivity towards human rights, universal and societal values, living beings, nature, and themselves.

### 6.1 Theoretical implications

The current research fills a gap in the literature by adopting a stakeholder perspective and investigating the contribution of implementing social marketing in the context of tourism to reduce poverty. To determine the role of tourism in poverty reduction, a phenomenological approach was first adopted in the study. Therefore, it is essential to establish the context in which patterns related to the subject can be formed. In this context, the dimensions of poverty, the effects of tourism on poverty, and the encountered barriers were determined through a phenomenological design by utilizing the participants’ experiences and associated thoughts. Furthermore, the components of social marketing in the context of tourism and its role in poverty reduction were determined using the grounded theory.

The study exhibits a comprehensive structure in terms of the methods used, the theories it relies on, and the results it presents. In the context of reducing poverty, the role of tourism through social marketing has been evaluated not only from an economic perspective but also in terms of its social, cultural, and psychological impacts on the target group, aligning with the adopted holistic approach. Furthermore, this holistic approach does not only focus on the positive effects that tourism can bring but also attempts to identify barriers and challenges. Thus, it aims to contribute to academic studies seeking solutions to poverty reduction in light of the barriers that need to be overcome. Moreover, due to the increased interest of academics in social issues and the multidimensional exploration of these issues, the research findings are expected to provide significant contributions to this goal. The study is approached within the context of behavior change and exchange theory, both based on the foundation of social marketing. In addition to the goal of creating behavior change, it has been noted that three of the five key components that Ramirez et al. (2017) identify in the social marketing approach, namely exchange theory, market segmentation, and marketing mix components, are related to the exchange theory and behavior change. Therefore, for social marketing in the context of poverty reduction through tourism, identifying the target audience, stakeholders, marketing mix, and the gains to be achieved through behavior change is crucial, offering a holistic perspective for future studies.

### 6.2 Practical implications

It is possible to evaluate social marketing implementations for the target group in the context of tourism. In this context, it is thought that the marketing mix elements put forward to reduce poverty through tourism will be guiding. It is possible to create behavioral changes in the target group by using social marketing mix elements. First of all, the product aimed at targeted behavior change can be tourist destinations, experiences, or tourist activities. In order to reduce poverty, touristic products or social responsibility projects can be created that will directly benefit local communities. The cost/price of the product that will encourage behavioral change is important in terms of accessibility of the product, the benefits it will offer to the target group and fair pricing. It will be possible to direct the target group to the product by delivering and/or promoting the product. In addition, the people mix element has been determined as the people who both offer the product and benefit from the product. It is possible to focus on the people mix element in determining the role of local communities and stakeholders within the tourism industry. While partnership expresses the stakeholders and duties and responsibilities specific to a social marketing program planned to be implemented, policies include inclusive policy makers/implementers in the social marketing program. Therefore, social marketing programs can be implemented to create desired behavior changes in children and young people, guide them towards achieving their dreams, setting goals for themselves, and preventing them from engaging in negative activities. With the support of the Provincial Directorate of Youth and Sports, trips and camps for children and young people can be organized. This way, it may be possible to prevent children and young people from acquiring any kind of negative habits (such as alcohol, tobacco, drugs, theft, etc.) due to the void they may find themselves in.

Tourism-based recreational activities that involve people with disabilities, especially disabled children, are suitable for a robust social marketing implementation. Within the scope of social policy, tourism businesses, local governments, disabled children, and their families should be considered as key stakeholders in such activities. This way, individuals with disabilities can adapt to society, and behavior changes towards disabled individuals in society can be fostered. Karaküçük (2012) mentioned that recreational resources and implementation methods can create recreational areas and behavior change in individuals. Additionally, research on individuals, families, and communities can be conducted to identify changes before and after the implementation of a social marketing program, address shortcomings, and ensure the program’s sustainability. In this context, sociologists, psychologists, educators, healthcare institutions, and their staff are also important stakeholders. However, due to concerns about the limitations that may arise from the private sector’s involvement in such activities, adopting a more comprehensive and sustainable social policy is a necessity.

Local governments are indeed important stakeholders due to their relationships with the local population. Bacak and Uzunay (2018) have emphasized that local governments play a crucial role in ensuring social participation, universal rights, equality, quality of life, and human resource development with the support of the central government. In this context, events organized by local governments in the context of tourism can help the local population understand tourism better. Through such experiences, it is possible to promote positive behaviors like making the most of one’s leisure time and reducing the likelihood of causing harm to oneself and the environment. Furthermore, social marketing initiatives can be developed to empower the local population, especially by supporting women in tourism entrepreneurship, economically empowering them, and facilitating their participation in social life. Similar support can also be extended to convicts. Support and opportunities such as providing materials to the target group, microcredit support, promoting cooperatives, and offering tax exemptions for tourism product production can be provided. To help the target group convert their skills and abilities into products, production factories can be established, workshops can be organized, and efforts can be made to reach products to tourists. Using online marketing tools and methods in addition to participant opinions within the scope of the promotion mix element aimed at reducing poverty through social marketing in the context of tourism can ensure that campaigns are adopted by wider groups. In this context, creating social media campaigns, sharing social responsibility projects, using content marketing, creating online collaborations and partnerships, creating/sharing interactive experiences, and using social media influencers and ambassadors will increase the effectiveness of social marketing programs. Additionally, online social marketing strategies will enable businesses in the tourism sector as a stakeholder to take an effective role in fighting poverty through social marketing.

### 6.3 Model development for poverty reduction through social marketing implementation in the context of tourism

Tourism is a sector with the potential to stimulate local economies. However, the negative impacts of tourism and shortcomings in its management can lead to increased poverty and social inequality among the local population. In this study, social marketing is proposed as a strategic tool for the sustainable and equitable development of the tourism sector. Within the context of tourism, through the model outlined in the study, which aims to create behavior change using social marketing to reduce poverty, the following benefits and opportunities are expected to emerge:

- *Skill Development and Education:* Training programs can be established with the aim of imparting skills related to the tourism sector to the local population (*target group*). Social marketing strategies can promote these programs and encourage participation (*social marketing mix*), enabling the local population to benefit from economic opportunities.
- *Increasing Local Awareness:* The contributions of tourism to the local economy and its positive impacts should be communicated to the *target group*. Through social marketing campaigns, it can be emphasized that when tourism is properly managed, it can provide job opportunities, income growth, and social development.
- *Preserving Cultural Values:* Through social marketing, local cultural values that may be negatively impacted by tourism activities can be protected and integrated harmoniously with tourism. Awareness among the *target group* can be achieved through engagement with *stakeholders*.
- *Support for Social Entrepreneurship:* The tourism sector (*stakeholders*) can create new business opportunities for local entrepreneurs (*target group*). Social marketing can provide support to the target audience in establishing their businesses by promoting sustainable tourism entrepreneurship.
- *Community Engagement/Collaboration and Empowerment:* Social marketing strategies should aim to actively involve local populations in the planning and implementation of tourism projects. This approach can empower the local population to play a more significant role in shaping the industry’s development. In this context, policies can promote fairer and more sustainable tourism practices by taking into account the needs and concerns of the *target group*.
- *Sustainable Tourism Development:* Social marketing can promote responsible and sustainable tourism practices that protect the environment and local culture, ensuring that tourism benefits the community in the long term.
- *Reduced Poverty:* Social marketing initiatives can focus on creating income-generating opportunities for the local population (*target group*), thus reducing poverty within the community.
- *Improved Quality of Life:* As the tourism sector thrives, it can lead to improvements in infrastructure, healthcare, and education, thereby enhancing the overall quality of life for the local population (*target group*).
- *Enhanced Social Equity:* Social marketing can address issues of social inequality by ensuring that the benefits of tourism are distributed more equitably among the local population (*target group*).
- *Tourist Satisfaction:* By promoting responsible tourism and highlighting the positive impact of their choices, tourists can have more satisfying and meaningful experiences, contributing to their understanding of the destination’s culture and community.

Understanding how social marketing strategies in the context of tourism can contribute to poverty reduction is a key element in creating behavior change within the target group. The success of social marketing campaigns relies on understanding the beliefs and attitudes of the target group towards tourism (Ajzen, 1991). The *Theory of Planned Behavior* assists in predicting individuals’ intentions and behaviors based on these intentions. In this study, the first step is to determine how the target group perceives tourism, how it can benefit them, what the drawbacks are, and how these challenges can be overcome to foster positive attitudes and behaviors. Subsequently, in the context of tourism, social marketing can help the target group understand environmental, social, and cultural norms, enabling them to shape their behaviors accordingly. The study suggests that social marketing implementations in the context of tourism can have a positive impact on poverty reduction. This can potentially increase the participation of the target group in tourism activities. Furthermore, the study highlights that providing education and skill development to the target group can boost their confidence and lead to behavior change. Therefore, social marketing can offer collaboration opportunities by emphasizing the involvement and contributions of the target group. Since individuals’ intentions and behaviors are shaped by their characteristics, social marketing strategies can convey tailored messages to the target audience through the marketing mix, demonstrating how tourism aligns with their specific needs and values.

The contribution of social marketing strategies to reducing poverty can be increased by providing mutual benefit, encouraging interaction and cooperation, using behavioral nudges, and creating emotional commitment, with the theory of exchange addressed by social marketing in the context of *Social Exchange Theory* of Homans (1958). The exchange theory inherently supports behavior change. In this context, the study emphasizes mutual benefit by highlighting the benefits and gains that social marketing in tourism can provide to the target group. Furthermore, by highlighting the economic opportunities that tourism can offer, the participation of the target group in tourism can be encouraged. Social marketing strategies can promote active involvement and collaboration among the target group. Positive experiences and gains related to tourism can be shared within the target group, influencing individuals and groups to have a more positive attitude towards tourism. In tourism, social marketing can utilize small incentives and facilitators (nudges), to increase local population participation. Social marketing strategies can also cultivate an emotional commitment to tourism within the target group.

As a result, this research presents important findings with common and/or different aspects (social, political, economic, and scientific) for policymakers, practitioners, and academics. A model has been developed based on the study’s results and the existing literature (Figure 1). In this context, the model can serve several functions. The model can be used for (a) segmenting the market by researchers/practitioners to achieve the behavior change that social marketing is based on, considering that social issues often encompass a broad audience, (b) selecting the target group from within the segmented market, (c) determining how and for what purpose researchers/practitioners will combine the elements of the marketing mix and which components need to be included based on the needs of the project/research, (d) identifying stakeholders for creating collaborations, (e) focusing on which dimensions of poverty should be addressed in developing behavior change and determining the roles of social marketing and tourism in this context, and (f) determining the benefits that the target group will gain through behavior change using these identified roles.

### 6.4 Limitations and future research

The findings and conclusions of the research are limited to the experiences, perceptions, thoughts, and values of the participants. In this context, one limitation of the study is the inability to include tourists (due to the necessity of addressing both domestic and foreign tourists separately and the potential language and cultural differences that may arise in interviews with foreign tourists). However, considering the plans for the presentation of touristic offers only from the supply side will cause the plans to fail. Therefore, research that includes tourists’ opinions is a necessity to consider the demand side of planning. Another limitation is that the research was predominantly conducted in a destination focused on winter tourism. Future studies can focus on differences shaped by the socio-cultural, economic, psychological structure, and ethical and individual values of different communities/destinations. In addition, the similarity/difference between the theoretical results of the research and the applications for the target group determined within the scope of the research can be revealed through social marketing programs prepared for the target group. Studies can be carried out to examine the behavior change that will occur as the target group accepts the cost in comparison to the benefits it will provide. In this context, research can be conducted to determine the course of action, present the results and benefits, and rectify errors and deficiencies in practice. Action research, a qualitative research design, can be used to conduct such studies.

## Data availability statement

The datasets presented in this article are not readily available because the dataset includes interviews with participants. It cannot be shared because it is private. Requests to access the datasets should be directed to tuba.gezen@atauni.edu.tr.

## Ethics statement

The studies involving humans were approved by Gazi University, Social Sciences Institute Ethics Committee. The studies were conducted in accordance with the local legislation and institutional requirements. The participants provided their written informed consent to participate in this study.

## Author contributions

TT: Writing – original draft, Conceptualization, Data curation, Investigation, Methodology, Software, Validation, Visualization. AH: Supervision, Writing – review & editing.
